# Vulcanizable Gutta-Percha in Dental Prosthesis

**Published:** 1904-09

**Authors:** J. Dunklin Reynolds

**Affiliations:** Marietta, Ga.


(Written for *The American Journal of Dental Science.*)

### VULCANIZABLE GUTTA-PERCHA IN DENTAL PROSTHESIS.

By J. Dunklin Reynolds, D. D. S., Marietta, Ga.

(Written for *The American Journal of Dental Science.*)

Why this material has so long escaped the notice or comment of those interested in prosthetic dentistry we fail to see.

After a careful perusal of the literature of the profession extending back for at least ten years we find very little mention of the procedure of applying rubber directly to the model in making artificial dentures: that is eliminating the process of first assembling the case in wax, flasking, separating, etc.

The text books and so called authorities fail to make any note of this method that we can find, though it offers a most fertile field for investigation and perfection.

The materials we use are as follows: vulcanizable gutta-percha and a solution of rubber made to a very *thick* creamy consistency, by dissolving the gutta-percha or any base plate rubber, in spirits of turpentine, which is used for coating the model; and a set of spatulas for molding and pressing the rubber base in place (our set consists of a double end, S. S. White wax spatula No. 2 cut in two and set in large wooden handles, also a larger spatula made from the handle of a table fork, which furnishes a larger instrument if needed, though some of the vulcanite serapes sold at the dental depots would answer quite as well for this purpose.)

After securing an accurate model and correct bite (without which no prosthetist should attempt to work) we proceed as follows: Taking for an illustration a full upper denture—we coat all surfaces, palatal, labial, and buccal, with the rubber solution mentioned above, using a small, rather stiff bristled brush to apply it, letting the coating of this solution extend a short distance beyond the surface to be covered by the rubber.

Allow this to rest a few minutes for the evaporation of what turpentine may remain on the surface. The model is then ready for the reception of the vulcanizable gutta-percha, which is superior to ordinary vulcanite rubbers for this special mode of application as it is much easier of manipulation—and furnishes as substantial and well appearing finished appliance. This material is cut in one large piece to conform in shape and size to the palatal surface of the model, which is warmed but *not burned* till thoroughly plastic and pressed to place with the fingers and ball of the thumb till absolutely in contact with the model, which if properly heated, it will readily do. The labial and buccal surfaces are built up the same way with strips and pieces, when the model will be ready for the reception of the teeth, which must be absolutely clean and free from all wax. Here we might say that if the gums of the patient are very conspicuous we use gum section teeth, if not, we use plain teeth, which we much prefer; but rarely ever use *pink rubber,* which really presents a worse and less artistic appearance after a short period of use than the red rubber highly polished and properly carved.

The teeth are warmed gently and pressed to their position in the rubber on the model and articulated as though you were using wax as a base plate.

After the teeth are in position and articulated the inequalities are filled in and made smooth with the gutta-percha and spatulas. the gums molded to shape, the case is then ready to invest, vulcanize and polish. As the case is not to be separated, the model can be invested in most any suitable metal box. wrapped with wire.

The S. S. White Dental Mfg. Co. furnish me with the following directions for vulcanizing vulcanizable gutta-percha: "From the time the mercury shows 212 degrees, allow thirty minutes to raise to 310 degrees and let it remain there for eighty minutes. When a very strong plate will be the result."

Where it is desirable pink rubber can be used with great ease by warming gently and placing in the exact position required without any subsequent fear of the red rubber coming through to the surface or of the tooth-pins becoming enveloped which is so often the case in closing the flask under pressure.

Clasps and bars for strengthening can be heated and put in position in the rubber with the assurance of finding them in place after opening, following vulcanization.

This method is readily adaptable to regulating appliances and offers a ready method of constructing interdental splints, facial and maxillary restorations, which are sometimes quite troublesome in flasking and separating to be packed with rubber as ordinarilly done.

For repairing this procedure is one of par excellence. The fractured plate is simply drawn into apposition; invested; the edges of fracture cut out and dovetailed or grooved; the surface painted with rubber solution; packed with gutta-percha, left smooth with a spatula and invested to be vulcanized, never opening the flask after the first investment until vulcanized.

Where it is desirable to try the plate in the mouth before vulcanizing, the rubber solution can be left off the model and continue as before; after the plate has been tried in the mouth, the articulation changed if necessary, the model can then be coated with the rubber solution, the plate warmed and returned to the model for vulcanization.

In mouths that are troublesome to fit it may be suggested that a base plate be made to accurately fit the mouth and the bite taken with this in position, on which the teeth are to be directly set and vulcanized.

To those unacquainted with the technique of working vulcanizable gutta-percha in this manner we would say, it will appear somewhat hard to handle at first but once mastered no other method will be used.

This process of working rubber was first called to the writer's notice in his father's (Dr. A. Reynold's) laboratory, where no other method of making rubber plates has been used for over fifteen years, with unusual success.

The features recommending this particular process over the commonly used method of separating and packing the flask for the introduction of the rubber consist principally in the accuracy of fit and elimination of the complications of opening and closing the flask under pressure with its attendant evil results.

The rugal and fine lines of the mouth do not suffer the change and obliteration necessarily the case in closing down a flask with pressure. Still plates made by the above described method show every *hairline* and are remarkable for their correct adaptation.

---

### A METHOD OF DUPLICATING A PLATE WITHOUT TAKING IMPRESSION OR BITE.

By Bessie B. Bennett, D. D. S.,
Baltimore, Md.

(Written for *The American Journal of Dental Science.*)

